# Importance of alexithymia on anxiety and depression in alopecia areata: a cohort study

**DOI:** 10.1093/skinhd/vzaf092

**Published:** 2026-02-04

**Authors:** Johan Fhager, Karin Örmon, Åke Svensson, Karin Sjöström

**Affiliations:** Department of Care Science, Faculty of Health and Society, Malmö University, Malmö, Sweden; The Västra Götaland Region Competence Centre on Intimate Partner Violence, Gothenburg, Sweden; Department of Health, Blekinge Institute of Technology, Karlskrona, Sweden; Department of Dermatology and Venereology, Skåne University Hospital, Malmö, Sweden; Department of Care Science, Faculty of Health and Society, Malmö University, Malmö, Sweden

## Abstract

**Background:**

Alopecia areata (AA) is an autoimmune hair loss disease, considered a psychosomatic disease with comorbid symptoms of depression and anxiety. Alexithymia, defined as difficulties in recognizing and describing feelings, has been found to be a vulnerability factor for developing anxiety and depression and somatic disease. The psychological burden of AA needs to be further investigated in larger studies by using standardized instruments developed to identify alexithymia, and clinical depression and anxiety. The outcome is important when treating patients with AA as well as for decisions on treatment.

**Objectives:**

To explore the prevalence of alexithymia and its subtypes and how they relate to depressive and anxiety symptoms in patients with AA.

**Methods:**

In this cohort study 100 patients with AA were interviewed about sociodemographic data, AA disease variables, and previous and present mental health. The Beck Depression Inventory-II (BDI-II), the Beck Anxiety Inventory (BAI) and the Toronto Alexithymia Scale-20 (TAS-20) were used to identify alexithymia, depression and anxiety. Associations between alexithymia and subtype scores – difficulties identifying feelings (DIF), difficulties describing feelings (DDF) and externally oriented thinking (EOT) – were analysed in relation to depression and anxiety scores. Relations between alexithymia, depressive and anxiety scores, and AA and sociodemographic variables were examined.

**Results:**

Prevalences of depression, anxiety and alexithymia in patients with AA were 16% (*n* = 16/100), 22% (*n* = 22/100) and 32% (*n* = 32/100), respectively. There was a statistically significant relation between DIF and anxiety and between DIF, DDF and depression. Lower levels of education were related to alexithymia, depression and anxiety symptoms. Alexithymia was statistically significantly more frequent among those who were younger at AA onset, in the relapsing form of AA and with nonfamilial AA. Previous mental affective illness was reported in 77% (*n* = 77/100) of patients with AA during the life course.

**Conclusions:**

Patients with AA had a higher prevalence of depression, anxiety and alexithymia compared with normative data. Alexithymia was found among those with earlier AA onset, younger age at interview and lower educational levels. Higher EOT scores were found among those with anxiety and lower education.

## Teaser

Patients with alopecia areata appear to be psychologically vulnerable, reporting higher levels of alexithymia, depression and anxiety. There are associations with the subtypes of alexithymia where depressive alopecia areata patients rate difficulties in both identifying and describing feelings, whereas patients with anxiety rate difficulties in identifying feelings and in externally oriented thinking. Information about the mental ill health in patients with alopecia areata is important for understanding the complexity of the disease as well in decision making about treatment options

What is already known about this topic?

- Alopecia areata (AA) is an autoimmune hair loss disorder associated with symptoms of depression and anxiety.
- The population prevalence of alexithymia, where individuals have difficulties recognizing and describing emotions, has in some studies been higher in patients with AA.
- Three subtypes of alexithymia have been proposed: difficulties in identifying emotions, difficulties describing emotions and a tendency to external thinking where feelings are expressed through the body.

What does this study add?

- Patients with AA reported high levels of alexithymia, depression and anxiety scores.
- Patients with AA who had higher depression scores also had higher alexithymia scores with difficulties in identifying and describing feelings, whereas patients with higher anxiety scores rated difficulties in identifying feelings and in externally oriented thinking.
- Information about mental health in patients with AA is important for understanding the burden of the disease, which in turn is important for decision-making about treatment options.

Alopecia areata (AA) is one of the most common hair loss diseases, often becoming chronic with unpredictable relapses leading to high disease burden. AA is suggested to be of autoimmune origin with psychosomatic features, meaning that psychological influences contribute to the disease.^1–7^ AA often starts with circular hair loss patches on the scalp, with a risk of progression to the loss of all scalp hair [alopecia totalis (AT)] and to a total loss of bodily hair loss [alopecia universalis (AU)].^1–3,8–10^ AA affects all age groups, genders and ethnicities, and has a lifetime prevalence of 0.58–2.1,^1,2,8–10^ with approximately 12% having a family history of the disease.^11^

The unpredictable nature of the disease is often experienced as devastating, such as spontaneous regrowth followed by anxiety-provoking relapses.^1,2,11^ The burden of AA involves impaired self-esteem and stigmatization.^12^ There has been a recent increase in research into the psychological symptoms of patients with AA, such as anxiety and depression, which generate various challenges in clinical dermatology.^2–7^ Whether there is an increased vulnerability among patients with AA needs to be further explored.

Some patients have greater difficulties with emotions related to their medical condition and use a concrete and bodily oriented way of thinking based on difficulties in expressing and describing feelings. This difficulty with identifying, describing and expressing emotions is seen as a vulnerability that has been named alexithymia, meaning ‘no words for feelings’.^13,14^ Alexithymia has been identified as playing a potential role in the clinical outcomes of psychosomatic disorders.^14–17^ Patients with these types of problems can be difficult to understand and identify because they have difficulties talking about their emotional problems, even if they have high cognitive functioning and are well educated.^15^ In addition, they may also have difficulties forming clinical alliances and respond poorly to treatment.^16,17^

Three subtypes of alexithymia have been identified: (i) difficulties identifying emotions and difficulties in distinguishing between emotions and bodily sensations; (ii) difficulties describing and communicating emotions to others; and (iii) an externally oriented style of thinking that predisposes individuals to being unable to cope with feelings internally or mentally, which may lead to the expression of feelings through bodily sensations.^13,14^ Alexithymia is recognized as an important psychological concept, considered as a vulnerability factor and a personality construct.^16–20^ Furthermore, alexithymia is suggested to play an important role in the development of many psychosomatic disorders,^14,16,17,20,21^ even in dermatological conditions such as AA,^6,7^ and to have an impact on autonomic, endocrine and immune activity, leading to a poor immune response that may increase the risk of developing other diseases.^21–23^ Alexithymia has been shown to be more common in patients with inflammatory dermatological diseases, such as severe atopic dermatitis (44%),^24^ psoriasis (28%)^25^ and vitiligo (24%),^26^ than in the general population (8–13%).^18^

One previous study found a negative relation between alexithymia and the level of education.^15^ There are further indications of a negative impact of alexithymia on AA treatments, including a lack of expressing own needs, wishes and emotional experiences,^16,17^ which may be of importance when different therapies are discussed, especially since the introduction of immunological therapies for AA.

Previous studies have found a relation between the alexithymia subtypes and depression and/or anxiety.^27,28^ The latter is often present in patients with AA and associated with worse clinical outcomes.^6,7^ How these subtypes are related to the psychological, sociodemographic and AA variables in patients with AA are, to date, not known. Three case–control studies with different methodologies have been performed to examine the relation between alexithymia and depression and/or anxiety; however, their results were conflicting.^29–31^

The relation between alexithymia, depression and anxiety in patients with AA still needs to be investigated in larger samples of patients, which stresses the need for further research. The present study aimed to examine the relation of alexithymia with anxiety and depression in a standardized manner. The prevalence of the alexithymia subtypes in patients with AA are still unknown and needs to be further explored, as does their association with depression, anxiety and AA disease variables.

## Patients and methods

### Participants and procedure

In total, 102 patients with AA were recruited consecutively: 60 patients from medical records (Melior^®^), 12 patients during regular visits and 30 patients via the recommendations of other patients to participate. Inclusion criteria were a duration of AA of at least 1 year, AA diagnosed by a specialist in dermatology [International Classification of Diseases, Tenth Revision (ICD-10)], ≥18 years of age and a good oral and written command of the Swedish language. Exclusion criteria included overt psychotic behaviour, intellectual disability, or if a person to be under the influence of alcohol or drugs. No patients were excluded. Two patients dropped out; one patient was advised against participation by a dermatologist because of a mental health condition, and another patient dropped out due to the study design. Finally, 90 women and 10 men with AA were included (*n* = 100).

All recruited patients received written and verbal information about the study before giving their consent to participate.

### Data and measures

The study procedure took place at a dermatology outpatient clinic. The interviewer (J.F.) was available during the procedure and conducted the structured interviews and self-assessment instruments. A study sample of 100 patients has previously been found to detect true relationships between variables.^32^

### Structured interview

The investigation started with a structured interview designed by two of the authors (J.F., K.S.). It contained questions about sociodemographics, medical background, comorbidities, medications and familial AA. Age at AA onset, duration, episodes and any hair regrowth were asked for ICD-10 diagnoses were used to grade the AA severity. In addition, questions about previous mental disorders and mental disorders in close vicinity to AA onset were asked. Information on substance abuse was collected. Alcohol consumption was identified by asking questions from the Alcohol Use Disorders Identification Test (AUDIT).^33^

Most answers were rated according to three- and five-grad scales. Some questions could be answered with ‘yes’ or ‘no’, with possibilities for expansion. Patients were then asked to complete three self-assessment instruments measuring alexithymia and symptoms of depression and anxiety.

### Self-assessment instruments

The Toronto Alexithymia Scale-20 (TAS-20)^13^ consists of 20 statements, divided into 3 subscales: difficulties identifying emotions [DIF; 7 items (7–35 points)]; difficulties describing emotions [DDF; 5 items (5–25 points)]; and the tendency to externally oriented thinking [EOT; 8 items (8–40 points)]. Each item is rated on a 5-point Likert scale. The total alexithymia score is the sum of responses to all statements (20–100 points), while the score for each subscale is the sum of subscale scores. A cut-off ≤51 = no alexithymia, 52–60 = possible or borderline alexithymia and scores ≥61 = alexithymia.^13^ Alexithymia can be looked upon as a continuum of difficulties in emotional interaction and communication.^34^ Hence, the two groups of ‘possible alexithymia’ and ‘alexithymia’ were merged into one alexithymia group in accordance with the merge of moderate and high groups of depression and anxiety.

The Beck Depression Inventory-II (BDI-II)^35^ consists of 21 statements about symptoms and attitudes, such as guilt, pessimism, self-loathing and various physical symptoms, experienced during the past 2 weeks. Each question is rated on a 4-point scale (0–3), with score 3 as the highest level of severity. The total score is obtained by adding the scores for the 21 questions (score range 0–63). Ranges of symptoms from 0 to 13 are considered minimal, 14–19 mild, 20–28 moderate and 29–63 as severe symptoms of depression.^35^ In this study, two groups were created: the ‘low depression group’, with minimal and mild depression scores; and the ‘depression group’, with moderate-to-severe depression scores, significant for having a clinical depressive state.^36^

The Beck Anxiety Inventory (BAI)^37^ consists of 21 statements about bodily reactions to anxiety and experiences of psychological fear. The statements are rated on a 4-point scale. Each question has the answer options ‘Not at all’, ‘A little’, ‘Some’ and ‘A lot’. Ranges of symptoms of anxiety are 0–7 (minimal), 8–15 (mild), 16–25 (moderate) and 26–63 (severe anxiety).^37^ Two groups were created: the ‘low anxiety group’, with minimal and mild anxiety scores; and the ‘anxiety group’, with moderate-to-severe anxiety scores, significant for clinical anxiety.^36^

### Statistical analyses

Statistical analyses were performed with SPSS version 28 (IBM, Armonk, NY, USA). Frequencies were given as numbers and percentages. The Kolmogorov–Smirnov test was used to assess the skewness of data, and to detect differences in variances between groups, the Levene's test was used. Total alexithymia score, and depression and anxiety scores were skewed. Correlations were calculated with the Spearman rank coefficient test. The Mann–Whitney *U* test and a Kruskal–Wallis test were used to examine the effect of education on participants’ reports of total alexithymia, DIF, DDF and EOT score. The TAS-20 subtype scores were normally distributed. The Student's *t*-test was used to test significance between normally distributed groups. Cross-tabulations with a χ2 test of 2 × 2 tables were used to analyse independent categorical data, and a Fisher's exact test was used for 2 × 3 tables and when cells had an expected count <5. A two-tailed *P*-value of <0.05 was considered significant.

## Results

The psychologicall, sociodemographic and AA variables of the study cohort are shown in Table 1. Median age at interview was 52.5 (range 18–93) years and age at AA onset was ≤25 years of age in 50% (*n* = 50) of the patients. Fifty-one per cent of the patients (*n* = 51) had a university degree and 80% (*n* = 80) were employed or retired. The majority described their economic situation as average or above average. Thirty-three per cent of the patients (*n* = 33) had moderate-to-high alcohol consumption (Table 1).

**Table 1:** Alexithymia in relation to psychosocial factors, and alopecia areata (AA) variables (*n* = 100)

Variables	Total	Alexithymia (*n* = 32)	No alexithymia (*n* = 68)	*P*-value
Gender				
Women	90	29	61	
Men	10	3	7	0.99
Marital status				
Married/cohabitants/in relationship	64	20	44	
Single/widow(er)	36	12	24	0.83
AA severitya (ICD-10)				
AA	60	22	38	
AT/AU	40	10	30	0.22
Progressive/relapsing type of AA				
Progressive	58	13	45	
Relapsing	42	19	23	0.02c
Number of AA episodes				
1–2	61	15	46	
≥3	39	17	22	0.39
Reported familial prevalence				
Yes	40	8	32	
No	60	36	24	0.04c
Education				
Low (compulsory school)	15	9	6	
Medium (upper secondary school diploma)	34	12	22	
High (university degree)	51	11	40	0.02^d^
Employment status				
Employed/studying/childcare leave	70	24	46	
Retirement	20	4	16	
Unemployed/sick leave	10	4	6	0.43
Self-estimated financial situation				
Low	20	7	13	
Average	49	19	30	
Above average	31	6	25	0.21
Amount of alcohol per occasionb				
None–low: 0–3 glasses	66	24	42	
Moderate–high: 4–6 glasses	23	5	18	
Above high: ≥7 glasses	11	3	8	0.21
Any abuse (drugs, food, gambling)				
None	76	22	54	
Any abuse	24	10	14	0.32

Data are presented as n. AA diagnosed according to the International Classification of Diseases, Tenth Revision (ICD-10). AA, alopecia areata; AT/AU, alopecia totalis/alopecia universalis.

^a^Measured according to the World Health Organization’s Alcohol Use Disorders Identification Test (AUDIT) (standard glass/occasion).

^b^Standard glass of wine or beer, 15 and 33–50 cl, respectively.

^c^χ2 test; Pearson χ2.

^d^χ2 test; Fisher’s exact test; test of significance: P < 0.05 (two-tailed).

Alexithymia scores did not differ between men and women. Contrary to expectations, no differences in depression and anxiety scores were found between men and women in this study, probably because of the small number of men who took part, which is why no division by gender was made (Table 2).

**Table 2:** Alexithymia in relation to anxiety and depressive score, and alopecia areata (AA) variables (n=100)

Variables	Total group	Alexithymia (*n* = 32)	No alexithymia (*n* = 68)	*P* -value
**Depressive score** (BDI-II)	8 (0–46)	15.5 (0–49)	5.0 (0–46)	<0.001^a^
**Anxiety score** (BAI)	7 (0–43)	11.5 (0–43)	5.5 (0–40)	<0.001^a^
Age at interview (years)	52.5 (18–93)	40.5 (18–78)	55.5 (25–93)	0.01^a^
Age at AA onset (years)	25 (0–78)	17.5 (5–78)	28.0 (0–64)	0.03^a^
AA duration (years)	17.5 (1–71)	18.0 (1–53)	16.0 (1–71)	0.09

Data are presented as median (range). BAI, Beck Anxiety Inventory; BDI-II, Beck Depression Inventory-II.

^a^Analyses performed with the Mann–Whitney U test.

### Alexithymia and alopecia areata

The prevalence of alexithymia was 32% (*n* = 32/100). Total TAS scores and TAS subgroup scores are presented in Table 3.

**Table 3:** Prevalence of alexithymia in the entire cohort and in younger vs. older patients (*n* = 100)

TAS scale/TAS subscales	Total cohort (*n* = 100)		Younger patients (*n* = 50)	Older patients (*n* = 50)	*P*-value
	Mean (SD)	Median (range)	Mean (SD)	Mean (SD)	
TAS-20 score	46.0 (15.96)	43.0 (22–90)	48.0 (16.10)	44.0 (15.73)	0.17^a^
DIF score	15.0 (6.69)	14.0 (5–31)	16.0 (6.66)	15.0 (6.75)	0.42^b^
DDF score	12.0 (5.22)	11.0 (4–31)	13.0 (5.74)	11.0 (4.51)	0.08^b^
EOT score	19.0 (15.96)	18.0 (8–42)	20.0 (6.80)	18.0 (6.86)	0.34^b^

Younger patients at AA onset were ≤ 25 years old; older patients at AA onset were > 25 years old. DDF, difficulties describing emotions; DIF, difficulties identifying emotions; EOT, externally oriented thinking; TAS-20, Toronto Alexithymia Scale-20. ^a^Mann–Whitney U test, two-tailed; ^b^Student’s t-test, two-tailed.

Within the alexithymia group, 22% (*n* = 22/100) had alexithymia and 10% (*n* = 10/100) had possible alexithymia. In patients with alexithymia, the onset of AA was 10.5 years earlier. Alexithymia was found to be statistically significantly higher in patients who were younger at AA onset (*P* = 0.3) and in patients who were younger at the time of interview (*P* = 0.01; Table 1). Younger patients reported more difficulties defining emotions and had a higher degree of EOT. The prevalence of alexithymia in the total sample and in younger vs. older patients is shown in Table 3. Alexithymia was statistically significantly more frequent in the relapsing form of AA and in patients with nonfamilial AA (Table 1).

A statistically significantly higher number of patients in the ‘no alexithymia’ group had progressive AA and reported a familial prevalence of AA (*P* = 0.04). No statistical relations were found between alexithymia and AA variables regarding severity of AA (AA, AT, AU) and number of episodes (Table 1).

### Depression, anxiety and alopecia areata

Depression and anxiety were found in 16% (*n* = 16/100) and 22% (*n* = 22/100) of patients with AA, respectively. Total mean (SD) depressive score was 10.3 (9.70) (median 8; range 0–46). Clinical depression was found in 16% where 10% (*n* = 10/100) had moderate depression and 6% (*n* = 6/100) had severe depression. Total mean (SD) anxiety score was 10.9 (10.46) (median 7; range 0–43). Clinical anxiety was found in 22% of patinets with AA, where 10% (*n* = 10/100) had moderate anxiety and 12% (*n* = 12/100) had severe anxiety. Depression and anxiety scores were higher in the younger group of patients with AA vs. the older group, but the comparisons did not reach statistical significance. When analysing questions about previous mental illness and mental illness in close vicinity to AA onset, 77% (*n* = 77/100) reported previous mental illness and 27% (*n* = 27/100) reported mental illness in close vicinity to AA onset. Most of the reported disorders belonged to the affective disorders (ICD-10); one patient described a reactive psychosis.

### Alexithymia, depression and anxiety

The correlations between scores for alexithymia and depression, alexithymia and anxiety, and depression and anxiety, were rho = 0.48, 0.46 and 0.71 (*P* < 0.001), respectively. Patients with alexithymia had statistically significantly higher depression and anxiety scores than patients with no alexithymia (Table 2). The relation between groups of depression, anxiety and alexithymia total scores and subtype scores are shown in Table 4.

**Table 4:** The relationship between the Toronto Alexithymia Scale-20 (TAS-20) subscales [difficulties identifying feelings (DIF), difficulties describing feelings, externally oriented thinking (EOT)], and depression and anxiety groups in patients with alopecia areata (*n* = 100)

TAS-20 subscale	Depression groups (BDI-II)			Anxiety groups (BAI)		
	Low depression (0–18) *n* = 84	Depression (19–63), *n* = 16	P-value	Low anxiety (0–16), *n* = 78	Anxiety (17–63), *n* = 22	*P*-value
	Mean (SD)	Mean (SD)		Mean (SD)	Mean (SD)	
DIF	14.0 (5.86)	21.6 (7.34)	<0.001^a^	13.9 (6.07)	19.8 (6.93)	<0.001^a^
DDF	11.1 (5.12)	14.9 (4.63)	0.006^a^	11.2 (5.37)	13.5 (4.24)	0.07^a^
EOT	18.4 (6.85)	22.0 (6.10)	0.053	18.2 (7.00)	21.6 (5.57)	0.04^a^

BAI, Beck Anxiety Inventory; BDI-II, Beck Depression Inventory-II. ^a^Student's *t*-test, two-tailed.

The lower the level of education, the higher the symptoms of anxiety and depression scores (Table 4). Patients with university degrees had statistically significant lower total TAS-20 alexithymia scores [χ^2^ (2,100) = 11.09 (*P* < 0.004)] compared with patients with compulsory school education and patients with upper secondary school education. When groups of the educational levels were compared according to the DIF, DDF and EOT subscales, higher scores were found in patients with lower educational level [EOT subscale χ^2^ (2,100) = 14.06 (*P* < 0.001) DDF subscale χ^2^ (2,100) = 7.78 (*P* = 0.02)]. The strongest statistically significant relationship was found for low compared with high education and the EOT subscale (Table 5).

**Table 5:** Relationship between education level and depression and anxiety groups and Toronto Alexithymia Scale-20 (TAS-20) and its subscales (*n* = 100)

	Educational level			*P*-value
	Low (*n* = 15)^a^	Medium (*n* = 34)^b^	High (*n* = 51)^c^	
BDI-II				
Low depression	8 (17)	29 (35)	47 (56)	
Depression	7 (44)	5 (31)	4 (25)	0.003^d^
BAI				
Low anxiety	8 (10)	26 (33)	44 (57)	
Anxiety	7 (32)	8 (36)	7 (32)	0.01^d^
TAS-20 score	54.0 (32–90)	43.0 (25–89)	39.0 (22–82)	0.004^d^
DIF score	17.0 (7–31)	14.5 (7–30)	13.0 (5–30)	0.12^d^
DDF score	15.0 (5–21)	12.0 (4–25)	9.0 (4–31)	0.02^d^
EOT score	24.0 (10–42)	18.5 (8–37)	17.0 (8–37)	<0.001^d^

Data are presented as n (%) or median (range). BAI, Beck Anxiety Inventory; BDI-II, Beck Depression Inventory-II; DDF, difficulties describing emotions; DIF, difficulties identifying emotions; EOT, externally oriented thinking. ^a^Low education: compulsory school. ^b^Medium education: upper secondary education. ^c^High education: university and college. ^d^anova performed with Kruskal-Wallis test, two-tailed.

## Discussion

The results of this study confirm a positive association between alexithymia and its subtypes, and depression and anxiety in patients with AA. The prevalence of alexithymia in patients with AA (32%) was 2.5–4 times higher vs. previous findings for general populations (8–13%).^18^ Recent prevalence data for alexithymia in patients with psoriasis and vitiligo indicate that alexithymia is more common in patients with AA.^25,26^ Only patients with severe atopic dermatitis seem to have a higher prevalence of alexithymia.^24^ Depressive and anxiety symptoms in patients with AA in this study were 3–4 times higher than normative data.^38–42^

To our knowledge, there is only one previous study, by Willemsen *et al*.,^15^ on patients with AA and their ratings on the different subscales for alexithymia when using the TAS-20. In comparison with the study of Willemsen *et al*.^15^ almost identical total TAS-20 scores and subscores were found in the present study. Patients in the depression group in this study showed difficulties in both identifying (DIF) and describing (DDF) emotions. Ths is consistent with a study on patients with depression by Li *et al*.,^27^ who found that they scored higher in the DIF and DDF subscales, but not in the EOT subscale. This finding is of importance as alexithymia may intensify depressive symptoms and has been shown to interfere with recovery.^18,19,43^

Alexithymia has previously been related to severe anxiety disorders such as panic disorders and generalized anxiety disorders, where higher scores were found on the DIF and DDF subscales vs. controls.^28^ We found that patients with AA in the anxiety groups were mostly troubled by difficulties in identifying feelings (DIF) and externalizing their feelings (EOT). This is in line with the psychodynamic theory of anxiety, which says that patients with anxiety disorders often are keen to place their psychological problems externally, in the shape of undefined external problems and catastrophic thinking. They also experience more stressful events in their surroundings that they are unable to cope with emotionally.^44,45^

In addition, over three-quarters of this study group had experienced one or more occasion of previous mental disorder. In about a third of patients, anxiety and/or depression coincided with the onset of AA. We did not specify the period of mental disorder in relation to AA onset but relied on the patient's answers, which is a weakness. However, in one previous study,^46^ where patients with AA were examined with the Diagnostic Interview Schedule (DIS),^47^ the results indicated one or more previous psychiatric diagnoses in 74% of cases.^46^

Alexithymia and its relation to anxiety and depression in patients with AA have, to our knowledge, only been examined in three previous case–control studies.^29–31^ None of these studies found a relation between alexithymia and depression. Sayar *et al*.^31^ found a positive relationship between trait anxiety (i.e. personality bound anxiety), measured by the Spielberger state-Trait Anxiety Inventory (STAI),^48^ and alexithymia. Cordan Yacizi *et al*.^29^ found a higher frequency of alexithymia in 43 patients with AA vs. control participants, but none for depression and anxiety. Sellami *et al*.^30^ found a significant relationship between anxiety and alexithymia only. However, they used an older version of the TAS (TAS-26) and used the Hospital Anxiety and Depression scale (HADS)^49^ to measure anxiety and depression.

For the abovementioned studies, control groups were not well-defined, especially regarding health and the risk of having alexithymia. The use of the HADS in these studies may be a problem when comparing results between the present study and two of the previous studies.^29,30^ The limited span of content in the HADS suggests a lower reliability and validity by oversampling, and lower discrimination to mild and moderate depression states,^50^ and although it is validated for screening, there are doubts over whether it should be used for research purposes.^50,51^ In comparison, the BDI-II^35^ and the BAI^37^ instruments used in the present study have been extensively studied, are considered to have a high reliability and are known to correspond well to clinical states of depression^52,53^ and anxiety.^54,55^

We did not use the alternative shorter version of the BDI-II for medical patients as patients with AA do not complain of physical symptoms such as itch, pain or skin sensations^12^ like patients with, for example, psoriasis.

Younger patients at AA onset had higher levels of alexithymia. Besides alexithymia, younger persons may also have more difficulties in processing traumas and negative emotional experiences because of less life experience and education, especially those younger than 25 years of age. In this study, all participants had higher scores in all TAS-20 subgroups, but the differences did not reach statistical significance. Furthermore, younger age at AA onset and lower quality of life have been reported in a previous study.^12^

An interesting relationship was confirmed between lower levels of education and alexithymia in patients with AA, as previously suggested by Willemsen *et al*.^15^ Subtype analyses showed that low EOT was most prominent among the highly educated. One possibility could be that education may have a protective effect on externalizing behaviour with a more reflective way of handling feelings. Difficulties in cognitive functioning have further been related to higher levels of alexithymia,^56^ difficulties that, in turn, may be related to a lower interest for education. In contrast, Sellami *et al*.^30^ did not find an association between educational level and alexithymia; however, they did not examine the TAS-20 subscales.

One strength of this study is the number of patients which, compared with other studies, can be considered the largest sample to date, increasing validity. Another strength is that patients were systematically interviewed by the first author (J.F.), a certified psychotherapist, who was present during the whole procedure. The use of validated and standardized instruments is considered a strength; however, their usefulness depends on the patient's ability to understand the items. Despite a large body of empirical evidence for use of the TAS-20,^57,58^ the specificity of the instrument has been questioned. Patients with alexithymia may not be able to assess their own deficiencies as they have difficulties recognizing the feelings described in the instrument.^59^ This may indicate that there is an underestimation of alexithymia in the study. This problem may also be present for the scoring of depression and anxiety. Because of the difficulties in understanding emotions,^59,60^ one approach to assessing alexithymia may be with the addition of a face-to-face interview.^61^ One alexithymia scale of interest asks for emotions of ‘feeling bad’ or ‘feeling good’ like the Perth Alexithymia Questionnaire (PAQ),^34^ which may enhance understanding for patients with alexithymia as they often experience their emotional state diffusely like ‘bad’ or ‘good’.^34,59^ We chose to add patients with possible or borderline alexithymia to the alexithymia group in accordance with the division of depression and anxiety into two groups and as the TAS-20 is a continuous scale of increasing levels of alexithymic severity as previously stated in the PAQ.^34^ A previous study showed that about 50% of those in the possible or borderline alexithymic group were considered to have alexithymia when measured by other instruments.^59^

Our study supports the suggestion that patients with AA suffer significant psychological morbidity. This should be considered and addressed as part of their care. Although emotional expression varies somewhat across cultures, individual identification of a basic affect has been shown to be universal,^62,63^ which is why our findings are likely to be generalizable, although there is a need for further research in different cultural areas.

Affect-focused evidence-based psychotherapies are now available to help patients with alexithymia, which makes clinical awareness of alexithymia even more important as alexithymia is related to a lower understanding of health conditions, and behaviours and responses to treatment and care.^16,17,20,43^ The higher prevalence of depression and anxiety in patients with AA may further stress this clinical importance.

Patients with AA reported higher scores of alexithymia, depression and anxiety vs. normative data. Alexithymia, and symptoms of depression and anxiety, should be taken into consideration at AA onset. Self-assessment instruments for alexithymia and affective disorders for patients with AA when visiting dermatology units are recommended. In particular, attention should be paid to patients who are young at AA onset or have lower level of education.

## Data availability

The data on which this article is based are available in the article, with some exceptions. These exceptions are necessary to protect the privacy of the patients who participated in the study.
